# A rare window for conservative extraction: learning from an asymptomatic thoracic nail gun injury

**DOI:** 10.1093/jscr/rjag169

**Published:** 2026-03-17

**Authors:** Muhammad Al-Ameen Alzetani, Aiman Alzetani

**Affiliations:** Faculty of Medicine, University of Southampton, Southampton SO16 6YD, United Kingdom; Cardiothoracic Surgery, University Hospital Southampton NHS Foundation Trust, Southampton SO16 6YD, United Kingdom

**Keywords:** penetrating thoracic injury, retained foreign body, nail gun injury, conservative management, case report, thoracic surgery

## Abstract

Penetrating thoracic injuries involving retained foreign bodies (RFBs) present a significant clinical challenge. While removal is generally indicated to prevent complications, invasive surgical approaches carry inherent risks. We present the case of a 60-year-old male who sustained an accidental nail gun injury to his left chest. Imaging revealed a 10 cm nail lodged in the lung, 3 mm from a branch of the pulmonary artery. Despite the proximity to a major vessel, the patient’s haemodynamic stability permitted a conservative approach. Under local anaesthesia and mild sedation, a prophylactic chest drain was inserted, and the nail was successfully extracted along its entry path. The patient recovered without incident and was discharged the following day. This case highlights that in select, stable patients, a carefully planned conservative extraction can be a safe and effective alternative to more invasive procedures like thoracotomy or video-assisted thoracic surgery, minimizing patient morbidity.

## Introduction

Penetrating thoracic injuries are uncommon but potentially life-threatening due to the proximity of vital structures, including major vessels and the heart. Retained foreign bodies (RFBs) can cause infection, haemorrhage, or further organ damage, and removal is generally indicated. However, the optimal approach depends on patient stability, the foreign body’s location, and available expertise. While thoracotomy and video-assisted thoracic surgery (VATS) provide safe visualization and removal, they carry risks of morbidity. We present a case of a retained intrathoracic nail adjacent to the pulmonary artery that was successfully managed with a conservative approach.

## Case report

**Figure 1:**
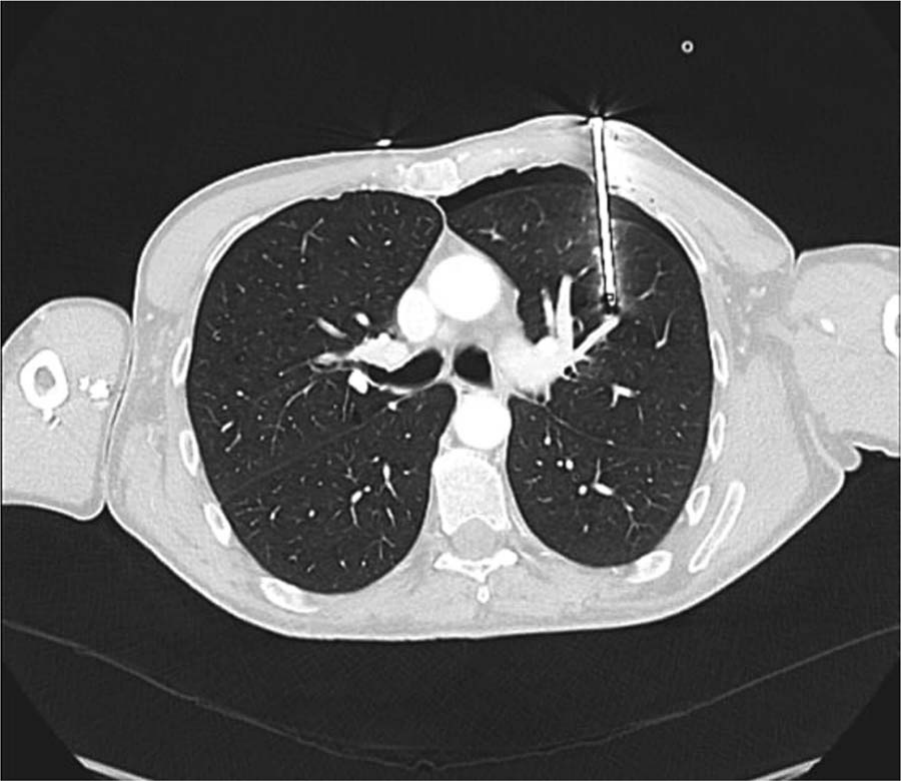
Axial CT chest demonstrating nail lodged in the left upper lobe, 3 mm from the anterior segmental branch of the pulmonary artery.

**Figure 2:**
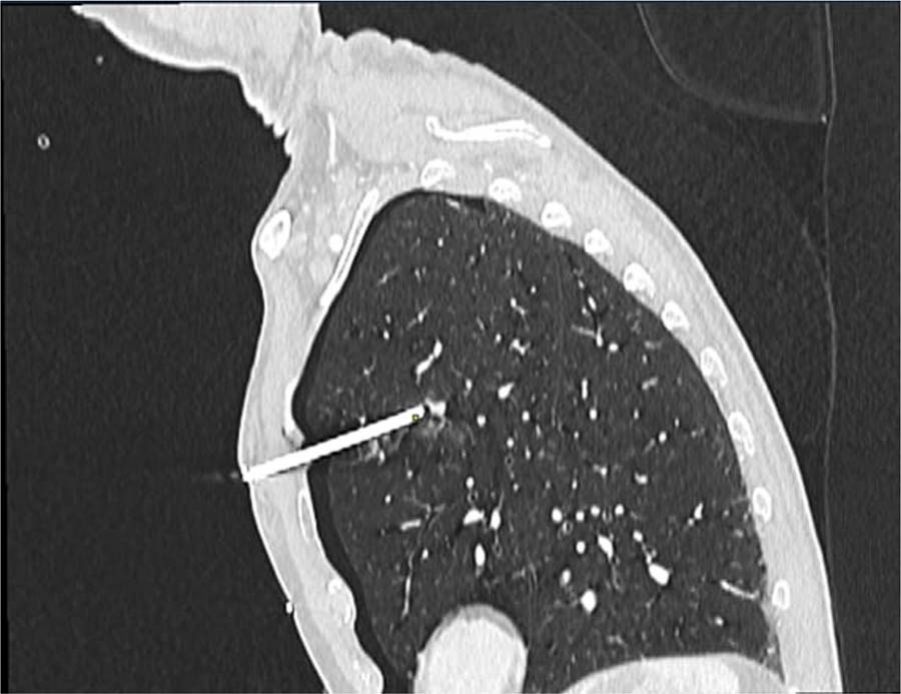
Sagittal CT chest demonstrating nail lodged in the left upper lobe, 3 mm from the anterior segmental branch of the pulmonary artery.

A 60-year-old male smoker presented following accidental discharge of a nail gun into his left chest. On arrival, he was asymptomatic with stable vital signs. Chest radiography showed a 10 cm nail within the left hemithorax and a small apical pneumothorax. Computed tomography (CT) imaging revealed the nail embedded in the upper lobe of the left lung, lying 3 mm from the anterior segmental branch of the pulmonary artery (Figs 1 and  2).

Given the risk of migration and potential vascular injury, invasive approaches including thoracotomy or VATS under general anaesthesia were considered. However, these carried risks of air leak exacerbation, tension pneumothorax, and nail displacement due to positive-pressure ventilation.

As the patient remained stable, a conservative plan was adopted. In the operating theatre, mild sedation and local anaesthetic infiltration were administered. The nail head was secured to prevent movement. A left chest drain was inserted under local anaesthetic into the safety triangle, releasing a small amount of air but no blood. The nail was then carefully extracted with forceps along its entry trajectory, and the wound was closed with interrupted sutures.

The patient remained haemodynamically stable throughout. Post-extraction chest radiography confirmed lung re-expansion and absence of haemothorax. He was monitored for 24 hours with repeat imaging and blood tests, which showed no further complications. The chest drain was removed, and he was discharged home.

## Discussion

Management of retained intrathoracic foreign bodies requires balancing the risks of removal against the morbidity of invasive surgery. Formal exploration via thoracotomy or VATS is often preferred in institutions with established trauma expertise, but both approaches increase postoperative pain, risk of infection, and length of stay.

In this case, the proximity of the nail to the pulmonary artery posed a theoretical risk of vascular injury during removal. However, careful conservative management — including chest drain insertion, maintenance of the extraction angle, and close monitoring — allowed safe removal without complications.

Published flowcharts from high-volume trauma centres, such as those in South Africa, suggest tailoring intervention to patient stability and imaging findings, ranging from simple extraction to full thoracotomy [1]. In less experienced centres, defaulting to invasive procedures may expose patients to unnecessary risk.

We propose that in selected stable patients, a rational, stepwise conservative strategy should be considered. This approach minimizes morbidity, avoids unnecessary thoracotomy, and can still escalate to surgery if deterioration occurs.
